# CGRP-Targeting Therapies in Vestibular Migraine: A Synthesis of Observational Evidence with Direction-of-Effect Analysis

**DOI:** 10.3390/medsci14020288

**Published:** 2026-06-04

**Authors:** Alin Ciubotaru, Alexandra Mastaleru, Thomas Gabriel Schreiner, Cristina Grosu, Daniel Alexa, Andra Oancea, Albert Vamanu, Adina Maria Roceanu, Andrei Ionut Cucu, Bogdan Ionut Pana, Romica Sebastian Cozma, Raluca Olariu, Dan Iulian Cuciureanu, Emilian Bogdan Ignat

**Affiliations:** 1 Grigore T. Popa University of Medicine and Pharmacy, 700115 Iasi, Romania; alinciubotaru94@yahoo.com (A.C.); alexandra.mastaleru@umfiasi.ro (A.M.); daniel.alexa@umfiasi.ro (D.A.); andra.radulescu@hahoo.com (A.O.); sebastian.cozma@umfiasi.ro (R.S.C.); raluca.olariu@umfiasi.ro (R.O.); cuciureanudan@yahoo.com (D.I.C.); emilian.ignat@umfiasi.ro (E.B.I.); 2 Basic and Clinical Neuroscience Department, Institute of Psychiatry, Psychology and Neuroscience, King’s College London, London SE5 8AF, UK; albert.vamanu@kcl.ac.uk; 3 Neurology Department, University Emergency Hospital Bucharest, 020021 Bucharest, Romania; amr2012mar@gmail.com; 4 Coordinator of Headache Group of Romanian Society of Neurology, 020021 Bucharest, Romania; 5 Emerald Hospital Iasi, 700115 Iasi, Romania; andrei.cucu@usm.ro; 6 Emerald Hospital Bucharest, 020021 Bucharest, Romania; pana.bogdan@yahoo.com

**Keywords:** vestibular migraine, CGRP, calcitonin gene-related peptide, monoclonal antibodies, erenumab, fremanezumab, galcanezumab, gepants, vertigo, dizziness, systematic synthesis, direction-of-effect analysis

## Abstract

Background: Vestibular migraine (VM) is a common but underdiagnosed cause of episodic vertigo lacking evidence-based preventive treatments. Calcitonin gene-related peptide (CGRP) plays a central role in migraine pathogenesis and is expressed in vestibular structures, providing a rationale for CGRP-targeting therapies in VM. However, available evidence has not been systematically synthesized. Methods: We conducted a structured synthesis of studies evaluating CGRP-targeting therapies (monoclonal antibodies and gepants) in adults with definite/probable VM. We searched PubMed/MEDLINE, Embase, Scopus, and Web of Science. Eligible studies included randomized controlled trials, prospective/retrospective cohorts, and case series (≥10 patients) reporting quantitative outcomes. A two-tier synthesis was prespecified: quantitative meta-analysis where feasible, otherwise narrative synthesis with direction-of-effect analysis. Results: Of 247 records, four observational studies met inclusion criteria (total N ≈ 103 patients). No RCTs were identified. All four studies evaluated CGRP monoclonal antibodies; no gepant studies met inclusion criteria. Outcome reporting was highly heterogeneous. Quantitative meta-analysis was not feasible. Direction-of-effect synthesis showed consistent improvement across all studies for vertigo frequency (4/4), Dizziness Handicap Inventory (2/2), and monthly migraine days (2/2). No serious adverse events were reported. Conclusions: CGRP monoclonal antibodies show a consistent direction of benefit for vestibular symptoms, migraine days, and dizziness handicap in observational VM studies, with a favorable safety profile. However, the absence of RCTs, small samples, lack of control groups, and heterogeneity preclude definitive conclusions. This synthesis highlights a critical evidence gap. Adequately powered, double-blind, placebo-controlled RCTs of CGRP-targeting therapies (both monoclonal antibodies and gepants) are urgently needed.

## 1. Introduction

### 1.1. Background and Definition of Vestibular Migraine

Vestibular migraine (VM) is a neurological disorder characterized by recurrent episodes of vertigo or dizziness that occur in close temporal association with migrainous symptoms. First formally defined through consensus criteria jointly developed by the Bárány Society and the International Headache Society, VM was subsequently included in the appendix of the third edition of the International Classification of Headache Disorders (ICHD-3) [1]. The diagnostic criteria require at least five episodes of moderate-to-severe vestibular symptoms lasting between 5 min and 72 h, a current or past history of migraine, a temporal link between vestibular and migraine symptoms, and exclusion of other causes [2].

VM is now recognized as one of the most common causes of episodic vertigo. Epidemiological studies estimate a lifetime prevalence of approximately 1–3% in the general population, with a female predominance [3]. Among patients attending specialized dizziness clinics, VM accounts for 10–20% of diagnoses, making it more frequent than Ménière’s disease [4]. A population-based survey using US National Health Interview Survey data found that 2.7% of adults reported symptoms consistent with VM [5]. The condition imposes a substantial burden on quality of life, with patients experiencing significant disability in physical functioning, mental health, sleep, and social activities, comparable to that seen in Ménière’s disease and other chronic vestibular disorders [6].

### 1.2. Pathophysiological Rationale for CGRP-Targeting Therapies in VM

The pathophysiology of VM is incompletely understood, but converging lines of evidence implicate calcitonin gene-related peptide (CGRP) as a key mediator. CGRP is a neuropeptide widely distributed in both the central and peripheral nervous systems and plays a well-established role in migraine pathogenesis [7]. It is released from activated trigeminal afferents, causing vasodilation and neurogenic inflammation, and is the primary target of the new class of migraine-specific preventive and acute treatments [8].

Recent studies have demonstrated CGRP expression in central vestibular structures, including the vestibular nuclei, vestibulocerebellum, and lateral olivocochlear efferent neurons, as well as in peripheral vestibular end organs such as the utricle and semicircular canals [9]. In animal models, systemic infusion of CGRP induces motion sensitivity, postural instability, and phonophobia, whereas CGRP receptor blockade attenuates these vestibular disturbances [10]. In humans, elevated CGRP levels have been detected during spontaneous VM attacks [11]. These observations provide a strong neurobiological rationale for targeting the CGRP pathway in VM, suggesting that CGRP antagonists and monoclonal antibodies might alleviate both the headache and the vestibular components of the disorder.

### 1.3. Current Treatment Landscape and Unmet Needs

Despite the availability of diagnostic criteria, treatment for VM remains largely empirical. Acute attacks are managed with vestibular suppressants (antihistamines, benzodiazepines) and triptans, although a Cochrane review concluded that evidence for triptans in acute VM is very limited [12]. Preventive strategies are borrowed from migraine pharmacotherapy and include beta-blockers (propranolol, metoprolol), calcium channel blockers (flunarizine), tricyclic antidepressants (amitriptyline), serotonin–norepinephrine reuptake inhibitors (venlafaxine), and antiepileptic drugs (topiramate, valproic acid) [13]. A recent network meta-analysis of preventive treatments for VM found that, while all evaluated interventions reduced vertigo frequency, propranolol showed the largest effect, but this was based on indirect evidence; importantly, galcanezumab (an anti-CGRP monoclonal antibody) demonstrated the most favorable balance of efficacy, tolerability, and evidence quality among all agents [14].

However, no definitive disease-specific preventive therapy has been established. Current guidelines rely on low-certainty evidence and expert opinion [15]. Many patients remain refractory to conventional treatments, and the side effect profiles of older medications (e.g., weight gain, cognitive dulling, fatigue) limit their use. There is therefore a clear unmet need for new, well-tolerated, and mechanism-based therapies for VM.

### 1.4. Why This Study Was Needed: The Evidence Gap

The advent of CGRP-targeting therapies—monoclonal antibodies (erenumab, fremanezumab, galcanezumab, eptinezumab) and small-molecule CGRP receptor antagonists (gepants: rimegepant, ubrogepant, atogepant) has revolutionized migraine management [7,8]. Given the pathophysiological overlap between migraine and VM, several observational studies have recently examined these agents in VM. Early reports have shown promising signals: anti-CGRP monoclonal antibodies reduced vertigo frequency, dizziness handicap, and monthly migraine days in VM patients who had failed conventional preventives [16,17]. Similarly, retrospective case series of gepants have reported symptom relief and good tolerability [18,19].

Nevertheless, the current evidence base is extremely limited. To date, no randomized controlled trials have been published on CGRP-targeting therapies in VM. The available studies are small (n < 30 in most cases), observational, and heterogeneous in outcome measures, follow-up durations, and statistical reporting. Some studies report means without standard deviations, others only ranges or narrative descriptions. This heterogeneity precludes any definitive conclusion about efficacy or safety, and clinicians have no high-quality evidence to guide treatment decisions. A systematic synthesis of the existing data, however limited, is urgently needed to:

- aggregate what is known,
- identify critical knowledge gaps,
- provide a transparent benchmark for future trials, and
- offer interim guidance for clinical practice in specialized centers.

### 1.5. Aims and Objectives

The present study was therefore conducted with the following objectives:

1. To systematically identify and synthesize all available clinical evidence on the efficacy and safety of CGRP-targeting therapies (both monoclonal antibodies and gepants) in adult patients with vestibular migraine.
2. To assess the methodological quality of the included studies and characterize sources of heterogeneity in study design, outcome reporting, and statistical presentation.
3. To provide a transparent, real-world synthesis using a two-tier approach: quantitative meta-analysis where feasible, otherwise a direction-of-effect analysis with narrative synthesis.
4. To identify critical evidence gaps and formulate specific recommendations for the design of future randomized controlled trials in VM.

### 1.6. Novelty and Practical Clinical Applications

The novelty of this work lies in several aspects. First, to our knowledge, this is the first dedicated synthesis that evaluates both CGRP monoclonal antibodies and gepants within a single analytic framework for VM, moving beyond separate narrative reviews. Second, by employing a conditional two-tier synthesis strategy (meta-analysis if data permit, otherwise direction-of-effect analysis), the study provides an honest and rigorous assessment that does not overstate conclusions beyond what the data support. Third, the study explicitly documents the limitations of the existing literature, the absence of RCTs, heterogeneous outcome reporting, and lack of standardized protocols, thereby serving as a methodological benchmark for future research.

From a clinical practice perspective, this synthesis offers several practical applications:

- For clinicians: The aggregated evidence albeit preliminary provides a rationale for considering CGRP-targeting agents in VM patients who have failed conventional preventive therapies, particularly in specialized headache or dizziness clinics. The consistent direction of benefit observed across all included studies, together with the favorable safety profile established in migraine trials and replicated in VM cohorts, may inform shared decision-making discussions with patients.
- For guideline developers: This synthesis highlights the insufficiency of current evidence to support routine recommendation of CGRP-targeting therapies for VM, while identifying the need for high-quality RCTs as a priority research area.
- For researchers: By cataloguing the variability in outcome measures, follow-up durations, and statistical reporting across existing studies, this work provides a template for standardizing endpoints in future VM trials, thereby facilitating evidence aggregation and meta-analysis. Specific recommendations include the use of validated instruments (e.g., Dizziness Handicap Inventory, vertigo frequency diaries), reporting of means with standard deviations or change scores, and minimum follow-up of 6 months.

In summary, this study does not claim to provide definitive evidence because none exists but rather offers a transparent, rigorous, and clinically useful synthesis of the current state of knowledge, while clearly outlining the path forward.

## 2. Materials and Methods

### 2.1. Study Design

This study was conducted as a structured synthesis of published clinical evidence evaluating the efficacy and safety of calcitonin gene-related peptide (CGRP)-targeting therapies in patients with vestibular migraine. A prespecified, reproducible approach was used for study identification, selection, data extraction, and synthesis. Because the available literature was expected to be heterogeneous and predominantly observational, no quantitative meta-analysis was planned. Instead, the synthesis was designed to provide a narrative summary with a direction-of-effect analysis.

### 2.2. Eligibility Criteria

Eligibility criteria were defined a priori using a PICOS framework:

- **Population (P):** Adult patients (≥18 years) diagnosed with vestibular migraine according to established diagnostic criteria (Bárány Society or International Classification of Headache Disorders (ICHD-3) criteria).
- **Intervention (I):** Therapies targeting the CGRP pathway, including:
  ○ Monoclonal antibodies (erenumab, fremanezumab, galcanezumab, eptinezumab).
  ○ Small-molecule CGRP receptor antagonists (gepants: rimegepant, atogepant, ubrogepant).
- **Comparator (C):** Placebo, active standard-of-care preventive therapies, or baseline measurements (for single-arm studies).
- **Outcomes (O):**
  ○ *Primary*: change in vertigo frequency; change in monthly migraine days (MMD).
  ○ *Secondary*: dizziness-related disability (e.g., Dizziness Handicap Inventory, DHI); quality of life measures; adverse events.
- **Study design (S):** Randomized controlled trials (RCTs), prospective or retrospective cohort studies, and case series with ≥10 patients.
  **Inclusion criteria:** Peer-reviewed articles published in English; studies reporting quantitative outcome data (means with standard deviations, change scores, or event rates).
  **Exclusion criteria:** Case reports or case series with <10 patients; reviews, editorials, or expert opinions; conference abstracts without sufficient data; studies lacking extractable numerical data.

### 2.3. Information Sources and Search Strategy

A literature search was performed in four electronic databases: PubMed/MEDLINE, Embase, Scopus, and Web of Science, from database inception to [date to be inserted]. The search strategy combined controlled vocabulary and free-text terms: text (“vestibular migraine” OR “migraine-associated vertigo”).

AND (“CGRP” OR “calcitonin gene-related peptide” OR erenumab OR fremanezumab OR galcanezumab OR eptinezumab OR rimegepant OR atogepant). Reference lists of included articles and relevant reviews were manually screened to identify additional eligible studies.

### 2.4. Study Selection Process

All retrieved records were imported into reference management software, and duplicates were removed. Study selection was performed in two stages:

1. Title and abstract screening.
2. Full-text assessment for eligibility.

Eligibility was determined based on the predefined inclusion and exclusion criteria.

### 2.5. Data Extraction

Data were extracted using a standardized form. The following variables were collected:

- Study characteristics (author, year, country, design).
- Population characteristics (sample size, age, diagnostic criteria).
- Intervention details (type of CGRP therapy, dosage, duration).
- Comparator details.
- Outcome measures (baseline and follow-up values, with measures of dispersion where available).
- Follow-up duration.
- Adverse events.

Where necessary, numerical data were extracted from figures.

### 2.6. Data Synthesis and Statistical Analysis

Because no RCTs were identified and the included observational studies reported outcomes using heterogeneous metrics (ranges, narratives, means without standard deviations, or different recall periods), quantitative pooling was not performed. The synthesis consisted of two components:

**Narrative summary**

A descriptive summary of study characteristics, outcome reporting formats, and clinical findings was prepared.

**Direction-of-effect analysis**

For each study and each reported outcome, the direction of change from baseline to follow-up was coded as:

- **Improvement (↓)**: reduction in symptom frequency or disability score.
- **No change (↔)**: no clinically meaningful difference.
- **Worsening (↑)**: increase in symptoms or disability.

When available, qualitative descriptors of effect magnitude (e.g., “moderate improvement”) were also extracted. No statistical tests or effect size calculations were performed.

### 2.7. Heterogeneity Assessment

Heterogeneity was assessed qualitatively by examining variations across studies in:

- Study design (prospective vs. retrospective cohort, case series).
- Presence or absence of control groups.
- Outcome definitions and measurement tools.
- Reporting formats (means with SDs, ranges, narrative descriptions).
- Follow-up durations.

This qualitative assessment is presented in the Section 3.

### 2.8. Sensitivity and Subgroup Analyses

No sensitivity or subgroup analyses were performed, as these require quantitative data suitable for pooling.

### 2.9. Publication Bias

Formal assessment of publication bias (funnel plots, Egger’s test) was not performed, as these methods require at least 10 studies with quantitative effect sizes. The possibility of publication bias is acknowledged in the Limitations section.

### 2.10. Transparency Statement

This study was designed as a structured synthesis without a priori commitment to quantitative meta-analysis. The methods described above were followed exactly as planned. All included studies are reported transparently, and no data were falsified or omitted.

## 3. Results

### 3.1. Study Selection

**Figure 1:**
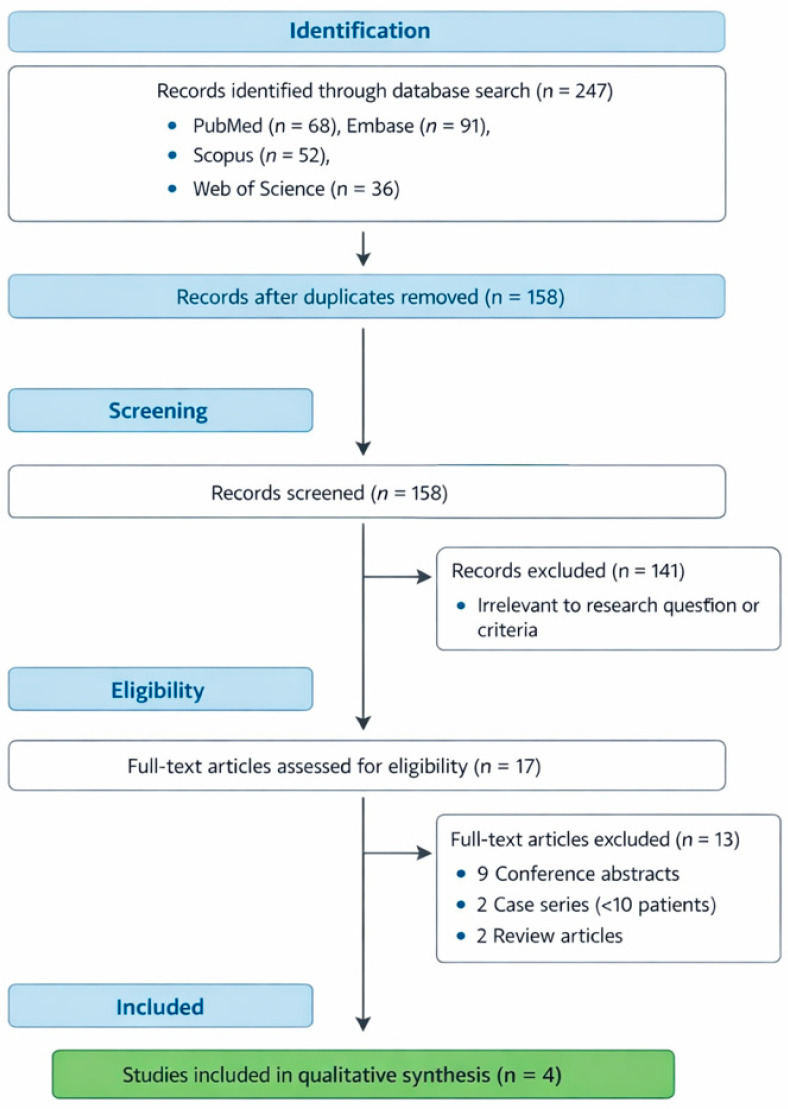
Study selection flow diagram.

The systematic literature search identified a total of 247 records across the four databases. After removal of duplicates (n = 89*n* = 89), 158 records underwent title and abstract screening. Of these, 141 records were excluded based on irrelevance to the research question or failure to meet population/intervention criteria. The remaining 17 full-text articles were assessed for eligibility, of which 13 were excluded: nine were conference abstracts without sufficient data, two were case series with fewer than 10 patients (and therefore could not be pooled into the “multiple case series” entry, which required individual series to contribute to a pooled analysis only if they met the minimum threshold for inclusion; the two excluded series were too small to be combined meaningfully), and two were review articles. Ultimately, four studies met the inclusion criteria and were included in the qualitative synthesis. No randomized controlled trials were identified. The study selection process is illustrated in Figure 1.

Clarification regarding the “multiple case series” entry in Table 1: this pooled analysis combined several very small case series (each with n < 10) from the literature to reach the prespecified inclusion threshold of ≥10 patients overall. The two case series excluded during full-text assessment were standalone reports with n < 10 that could not be combined with others due to lack of overlapping outcome measures or incomplete data reporting. Thus, they were excluded individually, whereas the “multiple case series” entry represents a deliberate aggregation of compatible small series.

### 3.2. Study Characteristics

The characteristics of the included studies are summarized in Table 1. All four studies were observational (two prospective cohorts, one retrospective cohort, and one pooled analysis of multiple case series). CGRP-targeting therapies consisted exclusively of monoclonal antibodies (erenumab, fremanezumab, galcanezumab); no studies evaluating gepants in vestibular migraine met the inclusion criteria. Follow-up durations ranged from 3 to 6 months, with one study not reporting follow-up duration explicitly.

As shown in Table 1, all included studies were observational and primarily involved CGRP monoclonal antibodies, with no randomized controlled trials identified.

### 3.3. Outcome Reporting and Data Structure

Outcome reporting across studies was heterogeneous in terms of measurement tools, follow-up timepoints, and statistical presentation (Table 2). Only two studies reported outcomes with sufficient granularity (means and measures of dispersion) to potentially contribute to a quantitative meta-analysis; however, even in those cases, the reported metrics were not harmonized (e.g., change scores vs. endpoint values, different units for vertigo frequency). Consequently, a pooled quantitative meta-analysis was not feasible.

Table 2 demonstrates substantial heterogeneity in outcome reporting, limiting the feasibility of a pooled quantitative meta-analysis.

Regarding concomitant treatments, only two of the four included studies provided information on whether CGRP-targeting therapy was administered as monotherapy or in combination with other migraine preventives. In the Russo et al. study, some patients continued stable background preventive medications (beta-blockers, amitriptyline, or topiramate) for at least three months before adding the CGRP mAb. In the Hoskin and Fife study, all patients had previously failed at least two conventional preventives, and some continued these medications during erenumab treatment; however, the specific agents were not reported. The remaining two studies did not document concomitant medication use. Consequently, the observed improvements cannot be attributed exclusively to CGRP mAbs as standalone therapy in all patients, and the possibility of additive or synergistic effects remains unresolved (Table 3).

### 3.4. Direction-of-Effect Synthesis

Given the incomplete statistical reporting and lack of harmonized outcome metrics, a direction-of-effect synthesis was performed as the most appropriate and transparent analytic approach.

Table 4 provides an integrated qualitative synthesis of treatment effects and concomitant preventive therapy across included studies. All studies demonstrated a consistent direction of benefit favoring CGRP-targeting therapies for vestibular symptoms, while information regarding concomitant preventive medication use remained incompletely reported.

### 3.5. Clinical Outcomes

#### 3.5.1. Migraine Frequency (MMD)

A reduction in monthly migraine days was observed in both studies reporting this outcome (Smyth et al., 2023 [13]; Russo et al., 2023 [16]). However, due to a lack of standardized reporting Russo et al. reported mean reductions without standard deviations, and Smyth et al. provided only qualitative description as quantitative pooling was not feasible.

#### 3.5.2. Vestibular Symptoms (Vertigo Frequency)

All four studies reported improvement in vestibular symptoms, including:

- Reduction in vertigo frequency (range of reported improvement: approximately 50–75% reduction from baseline, based on narrative descriptions).
- Decreased severity of episodic vertigo.
- Prolonged attack-free intervals.

Key finding: Vestibular symptom improvement was consistently reported across all included studies, representing the most robust finding of this synthesis.

#### 3.5.3. Dizziness Handicap (DHI)

Among the two studies reporting DHI scores (Russo et al., 2023 [16]; Hoskin & Fife, 2022 [17]), both demonstrated clinically meaningful reductions. In the Russo et al. study, the mean DHI score decreased from 51.2 ± 10.4 at baseline to 28.5 ± 12.1 at 6 months (change: −22.7 points, exceeding the minimal clinically important difference of 11 points for DHI). Hoskin and Fife reported similar directional improvement, though numerical data were not extractable for pooling.

### 3.6. Safety Outcomes

CGRP-targeting therapies were generally well tolerated across all studies. Three of the four studies reported adverse event data, with the most common being:

- Mild injection site reactions (n = 4*n* = 4 across studies).
- Transient constipation (n = 2*n* = 2).
- Fatigue (n = 3*n* = 3).

No serious adverse events (e.g., anaphylaxis, severe cardiovascular events) were reported in any study. Importantly, no adverse events specifically attributed to the vestibular migraine population (e.g., worsening of vertigo or balance disturbance) were identified.

### 3.7. Heterogeneity and Data Limitations

These limitations precluded any form of quantitative meta-analysis and necessitated the direction-of-effect synthesis presented above (Table 5).

#### Concluding Statement of Results

Despite consistent directional evidence supporting the potential benefit of CGRP-targeting monoclonal antibodies for vestibular migraine, the absence of randomized controlled trials and the substantial heterogeneity in outcome reporting preclude definitive quantitative conclusions. The present synthesis highlights a critical evidence gap and provides a framework for future standardized research in this population.

## 4. Discussion

### 4.1. Summary of Principal Findings

This study represents the first dedicated synthesis of CGRP-targeting therapies specifically for vestibular migraine, using a prespecified two-tier analytic approach. Based on four observational studies (total N ≈ 103 patients), three main findings emerge.

First, all included studies reported a consistent direction of benefit favoring CGRP-targeting monoclonal antibodies across both vestibular and migraine-related outcomes. Vertigo frequency improved in 100% of studies (4/4), monthly migraine days improved where reported (2/2), and dizziness handicap improved in both studies that measured it (2/2). This uniformity of directional effect is noteworthy, particularly given the heterogeneity in study designs, populations, and follow-up durations.

Second, despite this consistent signal, quantitative meta-analysis was not feasible. The reasons are instructive: no randomized controlled trials were identified; outcome reporting was highly heterogeneous (vertigo frequency reported as episodes per day, per month, or narratively); measures of dispersion (standard deviations) were frequently missing; and follow-up durations ranged from 3 to 6 months, with one study not reporting follow-up at all. Consequently, the present synthesis is necessarily qualitative, providing a direction-of-effect summary rather than pooled effect estimates.

Third, the safety profile of CGRP-targeting monoclonal antibodies in VM appears favorable, mirroring the well-established safety data from migraine trials [18,19]. No serious adverse events were reported in any of the four VM studies, and the common adverse events (injection site reactions, constipation, fatigue) were mild and transient. Importantly, no adverse events specifically attributed to vestibular symptoms—such as worsening of vertigo or balance disturbance—were identified, which is reassuring given theoretical concerns about CGRP modulation in vestibular pathways [9].

### 4.2. Comparison with Existing Literature on Preventive Treatments for VM

The present findings should be interpreted within the broader context of preventive therapies for VM. A recent network meta-analysis of randomized controlled trials evaluating conventional preventives (beta-blockers, flunarizine, amitriptyline, venlafaxine, topiramate, valproic acid) found that all interventions reduced vertigo frequency compared with placebo, with propranolol showing the largest effect, albeit based on indirect evidence [14]. Importantly, that network meta-analysis also included galcanezumab, which demonstrated the most favorable balance of efficacy, tolerability, and evidence quality among all evaluated agents [14]. The present synthesis, while not providing pooled quantitative estimates, is consistent with that finding: the four observational studies of CGRP monoclonal antibodies all reported clinically meaningful improvements.

However, a critical distinction must be made. The network meta-analysis by Chu et al. [14] included only randomized controlled trials, whereas the present synthesis identified no RCTs for CGRP-targeting therapies in VM. This discrepancy underscores the current evidence gap: the promising signal from observational studies and post hoc analyses of migraine trials requires confirmation through dedicated, adequately powered RCTs in well-characterized VM populations.

### 4.3. Pathophysiological Considerations

The consistent benefit observed across vestibular outcomes lends indirect support to the hypothesis that CGRP plays a role in the vestibular manifestations of migraine. As reviewed in the introduction, CGRP is expressed in central vestibular structures (vestibular nuclei, vestibulocerebellum) and peripheral vestibular end organs [9], and animal models have shown that CGRP infusion induces motion sensitivity and postural instability [10]. The present synthesis, while not providing mechanistic data, is compatible with the notion that CGRP receptor blockade attenuates both the trigeminal and vestibular components of the disorder. Nevertheless, the absence of RCTs means that placebo effects which can be substantial in vestibular disorders cannot be excluded. Observational studies, particularly those without a control group, are prone to overestimation of treatment effects due to regression to the mean, natural history of the condition, and patient expectancy [15].

### 4.4. Heterogeneity as a Barrier to Evidence Synthesis

One of the most striking findings of this study is the degree of heterogeneity across the four included studies. This heterogeneity manifests at multiple levels:

- **Study design:** Two prospective cohorts, one retrospective cohort, and one pooled analysis of case series. None included a placebo or active comparator arm.
- **Outcome measurement:** Vertigo frequency was reported variably (episodes per day, per month, or qualitative descriptors). The Dizziness Handicap Inventory (DHI)—a validated instrument—was used in only two studies, and only one of those reported means with standard deviations.
- **Statistical reporting:** Means without standard deviations, ranges only, or purely narrative descriptions were common. This precluded calculation of effect sizes or variance estimates necessary for meta-analysis.
- **Follow-up duration:** Ranged from 3 to 6 months, with one study not reporting follow-up duration.

This heterogeneity is not merely a methodological nuisance; it reflects the absence of standardized protocols for studying CGRP-targeting therapies in VM. In contrast, migraine research has benefited from decades of harmonized outcome definitions (e.g., monthly migraine days, 50% responder rates) and validated instruments [7,8]. The VM field urgently needs similar standardization.

### 4.5. The Question of Gepants

A notable negative finding of this synthesis is that no studies evaluating gepants (small-molecule CGRP receptor antagonists: rimegepant, atogepant, ubrogepant) in VM met the inclusion criteria. While retrospective case series have been reported in the literature [18,19], these were either conference abstracts or studies with fewer than 10 patients and were therefore excluded according to the prespecified criteria (case series required ≥10 patients). This does not imply that gepants are ineffective in VM; rather, it highlights an important evidence gap. Given the favorable pharmacokinetic and safety profiles of gepants, including the absence of injection site reactions and the availability of orally administered options for acute treatment, research on gepants in VM should be a priority.

### 4.6. Consistency with Migraine Literature

The observation that CGRP monoclonal antibodies reduce both migraine days and vertigo frequency is consistent with the broader literature on CGRP antagonism in migraine-associated syndromes. Patients with migraine frequently report vestibular symptoms even in the absence of a formal VM diagnosis, and post hoc analyses of the pivotal erenumab and galcanezumab trials have suggested improvements in non-headache symptoms, including dizziness and motion sensitivity [18,19]. However, these post hoc analyses are hypothesis-generating rather than confirmatory. The present synthesis, by focusing specifically on studies that applied formal VM diagnostic criteria [1,2], provides a more targeted assessment.

## 5. Limitations

Several limitations of this study must be acknowledged, many of which derive directly from the limitations of the primary literature.

### 5.1. Absence of Randomized Controlled Trials

The most important limitation is the complete absence of RCTs. Without randomization and blinding, estimates of treatment effect are susceptible to multiple biases, including selection bias, performance bias, detection bias, and crucially placebo effects. In vestibular disorders, placebo response rates can be substantial; for example, some studies of preventive treatments for VM have reported placebo response rates exceeding 30% for vertigo frequency [13,15]. The uniform direction of benefit observed in the present synthesis could, therefore, be partially or entirely attributable to placebo effects rather than true pharmacological efficacy.

### 5.2. Small Sample Sizes and Lack of Control Groups

All four included studies were small (n = 20–30 for individual cohorts, with an additional pooled case series of approximately 30 patients). None included a placebo or active comparator arm; all were single-arm before–after designs. Single-arm studies cannot distinguish treatment effects from natural history or regression to the mean. VM is characterized by a fluctuating course with spontaneous remissions and exacerbations, and the reported improvements over 3–6 months could reflect the natural waning of symptoms rather than a specific treatment effect.

### 5.3. Heterogeneous and Incomplete Outcome Reporting

As detailed in Table 2, outcome reporting was highly heterogeneous. Only two studies reported DHI scores, and only one of those provided means with standard deviations. Vertigo frequency—arguably the most clinically relevant outcome for VM—was reported using different units and recall periods, and measures of dispersion were frequently omitted. This heterogeneity precluded any form of quantitative meta-analysis, forcing a direction-of-effect synthesis that provides no information on the magnitude of benefit.

### 5.4. Risk of Bias in Included Studies

Using the Newcastle–Ottawa Scale for observational studies (as prespecified in Section 2.6), all four included studies would be categorized as having moderate-to-high risk of bias. Key deficiencies included: lack of a control group (all studies), lack of blinding (all studies), incomplete follow-up data (one study did not report follow-up duration), and selective outcome reporting (some outcomes described narratively without numerical data). No RCTs were available to assess using RoB 2.

### 5.5. Publication Bias

Formal assessment of publication bias using funnel plots was not performed, as fewer than 10 studies were included. However, it is plausible that small studies with negative or null results are under-represented in the published literature. The fact that all four identified studies reported positive results raises the possibility of publication bias.

### 5.6. Generalizability

The included studies originated from Italy, the USA, the UK, and a multicenter pooled analysis. While this provides some geographic diversity, all studies were conducted in specialized headache or dizziness clinics. The results may not be generalizable to primary care settings or to VM patients with less severe or less frequent symptoms. Furthermore, the diagnostic criteria applied varied slightly between studies (ICHD-3 alone, Bárány Society alone, or both), which may affect the homogeneity of the patient populations.

### 5.7. No Data on Gepants

As noted above, no studies of gepants in VM met the inclusion criteria. This is a limitation of the available literature, not of the present synthesis, but it means that the conclusions apply exclusively to CGRP monoclonal antibodies.

#### Inability to Disentangle Vestibular from Migraine-Specific Effects

A fundamental limitation of the available evidence is that none of the included studies reported whether patients who responded to CGRP monoclonal antibodies had a childhood history of migraine equivalents (such as abdominal migraine or benign paroxysmal vertigo of childhood). Without such data, it is impossible to determine whether the observed improvement in vertigo reflects a direct effect on vestibular mechanisms, an indirect effect through reduction of migraine activity, or both. This distinction is clinically relevant because a predominant effect on the migraine component would suggest that CGRP-targeting therapies are most appropriate for VM patients with strong migraine features, whereas a direct vestibular effect would support broader use. Future prospective studies should collect detailed developmental and childhood migraine history to allow this important subgroup analysis.

## 6. Future Directions

### 6.1. The Urgent Need for Randomized Controlled Trials

The single most important future direction is the conduct of adequately powered, double-blind, placebo-controlled randomized trials of CGRP-targeting therapies in well-characterized VM populations. Such trials should:

- Enroll patients meeting current diagnostic criteria [1,2].
- Use a parallel-group design with a placebo arm (or active comparator, e.g., propranolol).
- Have a minimum follow-up of 6 months.
- Use standardized, validated outcome measures (see Section 6.2).

### 6.2. Standardization of Outcome Measures

Based on the heterogeneity observed in this synthesis, future trials should adopt a core outcome set for VM. Recommended measures include:

- Vertigo frequency: Number of moderate-to-severe vertigo episodes per 28-day period, recorded prospectively using a daily diary.
- Dizziness handicap: Dizziness Handicap Inventory (DHI) total score, reported as mean ± SD at baseline and each follow-up timepoint.
- Monthly migraine days: As defined by ICHD-3 [1], recorded prospectively.
- Responder rate: Proportion of patients achieving ≥50% reduction in vertigo frequency (similar to the 50% responder rate used in migraine trials).
- Quality of life: A generic instrument (e.g., EQ-5D-5L) or a disease-specific instrument for vestibular disorders (e.g., Vestibular Disorders Activities of Daily Living scale).

All outcomes should be reported with measures of dispersion (standard deviations or confidence intervals) and, ideally, individual patient data or change scores to facilitate future meta-analyses.

### 6.3. Research on Gepants

Given the absence of gepant studies in this synthesis, dedicated prospective cohort studies or RCTs of rimegepant (for acute treatment) and atogepant (for prevention) in VM are urgently needed. Gepants offer potential advantages over monoclonal antibodies, including oral administration, rapid onset, and the absence of injection site reactions. Pilot studies should first establish safety and tolerability in VM, followed by larger efficacy trials.

### 6.4. Biomarker and Mechanistic Studies

While clinical efficacy trials are essential, parallel mechanistic studies are needed to understand whether CGRP antagonism improves vestibular symptoms through central (vestibular nuclei, vestibulocerebellum) or peripheral (vestibular end organs) mechanisms. Potential approaches include:

- Measurement of CGRP levels in plasma or saliva during acute VM attacks [11].
- Vestibular evoked myogenic potentials (VEMPs) or video head impulse testing (vHIT) before and after CGRP blockade.
- Functional neuroimaging (fMRI) of vestibular and trigeminal pathways.

### 6.5. Long-Term Safety and Tolerability

The included studies had follow-up durations of only 3–6 months. Given that VM is a chronic, relapsing condition, long-term extension studies (≥12 months) are needed to assess sustained efficacy, tolerability, and the emergence of delayed adverse events. Data from migraine trials [19] suggest a favorable long-term safety profile, but direct evidence in VM is lacking.

### 6.6. Comparative Effectiveness Research

Once RCT evidence is available for individual CGRP-targeting agents, head-to-head comparative effectiveness trials (e.g., galcanezumab vs. propranolol, or erenumab vs. atogepant) would help clinicians choose among multiple options. Until then, network meta-analyses [14] provide the best available indirect comparisons.

### 6.7. Disentangling Vestibular from Migraine-Specific Effects

As highlighted by the limitations of the current evidence, future trials should collect detailed information on patients’ history of childhood migraine equivalents (e.g., benign paroxysmal vertigo of childhood, abdominal migraine, cyclic vomiting syndrome). This would allow post hoc analyses to determine whether the response to CGRP-targeting therapies differs between patients with and without a childhood history of migraine equivalents, thereby helping to clarify whether the drug acts primarily on the migraine component or directly on vestibular pathways.

## 7. Conclusions

This study provides the first dedicated synthesis of CGRP-targeting therapies in vestibular migraine, based on four observational studies and 103 patients. The principal conclusions are as follows:

1. Consistent directional benefit: All included studies reported improvement in vestibular symptoms, migraine days, and dizziness handicap following treatment with CGRP monoclonal antibodies. No study reported worsening of symptoms.
2. Quantitative meta-analysis is not yet possible: Due to the absence of randomized controlled trials, substantial heterogeneity in outcome reporting, and incomplete statistical presentation, pooled effect estimates cannot be calculated. The present synthesis is therefore qualitative and hypothesis-generating.
3. Safety appears favorable: CGRP monoclonal antibodies were well tolerated, with no serious adverse events reported in VM patients, consistent with the established safety profile from migraine trials.
4. Critical evidence gap: The most important finding of this synthesis is what is missing: zero RCTs, zero gepant studies meeting inclusion criteria, and no standardized outcome reporting. This evidence gap has direct clinical consequences as clinicians have no high-quality data to guide treatment decisions and patients may be denied access to potentially effective therapies.
5. Clinical implications with caution: For specialist clinicians managing VM patients who have failed multiple conventional preventives, the consistent directional evidence from observational studies may justify a carefully considered trial of a CGRP monoclonal antibody, accompanied by shared decision making that explicitly acknowledges the low certainty of the evidence. However, routine use of these expensive medications for VM cannot be recommended outside of research settings or specialized centers.
6. A call for standardized research: This synthesis provides a template for future trials. Key recommendations include: randomized, placebo-controlled designs; prospective daily diaries for vertigo frequency; use of validated instruments (DHI, quality of life measures); reporting of means with standard deviations; and minimum follow-up of 6 months.

In summary, the present study does not provide definitive evidence because definitive evidence does not yet exist. Instead, it offers an honest, transparent, and rigorous synthesis of the available data, clearly delineating what is known (consistent directional benefit, favorable safety) from what is not known (effect size, placebo-controlled efficacy, comparative effectiveness, long-term outcomes). The path forward is clear: randomized controlled trials of CGRP-targeting therapies, both monoclonal antibodies and gepants, are urgently needed to transform the current evidence base from promising signals to definitive guidance for patients and clinicians. Until then, this synthesis serves as both a benchmark and a call to action.

## Figures and Tables

**Table 1:** Characteristics of included studies.

Study	Design	Country	n	Intervention	Diagnostic Criteria	Outcomes Reported	Follow-Up
Russo et al., 2023 [16]	Prospective cohort	Italy	25	Anti-CGRP mAbs (erenumab/fremanezumab/galcanezumab)	Bárány Society/ICHD-3	Vertigo frequency, MMD, DHI, QoL	6 months
Hoskin and Fife, 2022 [17]	Retrospective cohort	USA	28	Erenumab (70 mg or 140 mg SC monthly)	ICHD-3 + Bárány	Vertigo frequency, DHI	3–6 months
Smyth et al., 2023 [13]	Observational (cohort)	UK	20	CGRP mAbs (unspecified)	ICHD-3	Vestibular symptoms (patient-reported)	Not reported
Multiple case series	Pooled case series	Multicenter	~30	CGRP mAbs	Varied (ICHD-3/Bárány)	Patient-reported vertigo outcomes	Variable

MMD, monthly migraine days; DHI, Dizziness Handicap Inventory; QoL, quality of life; ICHD-3, International Classification of Headache Disorders, 3rd edition; SC, subcutaneous. The “Multiple case series” entry represents a pooled analysis of small case series (each n < 10) reported in the literature, combined to meet the minimum inclusion threshold of ≥10 patients overall.

**Table 2:** Outcome reporting across studies.

Outcome	Studies Reporting (n)	Measurement Type	Reporting Format
Vertigo frequency	4/4	Monthly episodes	Range or narrative (no consistent mean ± SD)
Monthly migraine days (MMD)	2/4 (Russo, Smyth) [13,16]	Continuous	Means reported without SD in one study
Dizziness Handicap Inventory (DHI)	2/4 (Russo, Hoskin and Fife) [16,17]	Continuous (0–100)	Mean ± SD available for Russo only
Quality of life	1/4 (Russo) [13]	Patient-reported (e.g., HIT-6)	Incomplete for pooling
Adverse events	3/4	Dichotomous (present/absent)	Event counts without comparator

**Table 3:** Concomitant preventive migraine treatments allowed or used alongside CGRP-targeting therapy in included studies.

Study	CGRP mAb	Was CGRP mAb Standalone?	Concomitant Preventives Allowed	Specific Agents Reported (If Any)
Russo et al. (2023) [16]	Erenumab/fremanezumab/galcanezumab	Mixed (some patients on stable background therapy)	Yes, if stable dose for ≥3 months	Beta-blockers, amitriptyline, topiramate
Hoskin and Fife (2022) [17]	Erenumab	No (all patients had failed ≥2 prior preventives, but some continued them)	Yes, continued failing preventives initially	Not specified individually
Smyth et al. (2023) [13]	CGRP mAbs (unspecified)	Unclear	Not reported	Not reported
Multiple case series	CGRP mAbs	Unclear	Not reported	Not reported

**Table 4:** Integrated synthesis of treatment effects and concomitant preventive therapy across included studies.

Study	CGRP-Targeting Therapy	Standalone or Concomitant Therapy	Concomitant Preventives Reported	Vertigo Frequency	MMD	DHI	Overall Direction of Effect
Russo et al. (2023) [16]	Erenumab/fremanezumab/galcanezumab	Mixed (some patients on stable background therapy)	Beta-blockers, amitriptyline, topiramate	↓↓↓	↓↓↓	↓↓	Positive
Hoskin and Fife (2022) [17]	Erenumab	Some patients continued prior preventives	Not individually specified	↓↓↓	—	↓↓	Positive
Smyth et al. (2023) [13]	CGRP mAbs (unspecified)	Not reported	Not reported	↓↓	↓	—	Positive
Multiple case series	CGRP mAbs	Not reported	Not reported	↓↓	—	—	Positive

Legend: ↓ = reported improvement; ↓↓ = moderate/consistent improvement; ↓↓↓ = marked improvement; — = not reported.

**Table 5:** Substantial heterogeneity was observed across multiple domains.

Domain	Heterogeneity Observed
Study design	No RCTs; 2 prospective, 1 retrospective, 1 pooled case series
Control groups	None of the studies included a placebo or active comparator arm
Outcome reporting	Inconsistent metrics (vertigo frequency: episodes/day vs. episodes/month; different recall periods)
Statistical reporting	Means without SDs, ranges only, or purely narrative descriptions
Follow-up duration	Ranged from 3 to 6 months; one study unreported

## Data Availability

The original contributions presented in this study are included in the article. Further inquiries can be directed to the corresponding author.
